# Advances in Bioactive Compounds and Cancer Therapy—Insights from the Special Issue “Bioactive Compounds in Cancers”

**DOI:** 10.3390/ijms26125632

**Published:** 2025-06-12

**Authors:** Hassan Bousbaa, Honorina Cidade, Patrícia M. A. Silva

**Affiliations:** 1 UNIPRO—Oral Pathology and Rehabilitation Research Unit, University Institute of Health Sciences (IUCS-CESPU), 4585-116 Gandra, Portugal; hassan.bousbaa@iucs.cespu.pt (H.B.); hcidade@ff.up.pt (H.C.); 2 Laboratory of Organic and Pharmaceutical Chemistry, Department of Chemical Sciences, Faculty of Pharmacy, University of Porto, Rua de Jorge Viterbo Ferreira 228, 4050-313 Porto, Portugal; 3 CIIMAR/CIMAR LA, Interdisciplinary Centre of Marine and Environmental Research, University of Porto, Terminal de Cruzeiros do Porto de Leixões, 4450-208 Matosinhos, Portugal; 4 Associate Laboratory i4HB, Institute for Health and Bioeconomy, University Institute of Health Sciences—CESPU, 4585-116 Gandra, Portugal; 5 UCIBIO—Applied Molecular Biosciences Unit, Translational Toxicology Research Laboratory, University Institute of Health Sciences (1H-TOXRUN, IUCS-CESPU), 4585-116 Gandra, Portugal

Cancer remains one of the most critical global health challenges, being responsible for millions of deaths each year and exhibiting a rising incidence worldwide. Projections estimate that the number of new cancer cases will increase by approximately 49.6% between 2020 and 2040, rising from about 19.98 million to nearly 29.89 million annually [1]. This growing burden not only reflects the persistent nature of the disease but also highlights the urgent need for more effective, safe, and accessible treatment modalities. At the core of this challenge lies the biological complexity of cancer. Tumor heterogeneity, both between and within patients, combined with genetic and epigenetic variability, results in diverse tumor behaviors and unpredictable responses to therapy [2]. Moreover, resistance to conventional treatments such as chemotherapy, radiotherapy, and even some targeted therapies remains a major hurdle. This resistance often emerges through dynamic tumor evolution, including clonal selection, tumor microenvironment adaptation, and the activation of compensatory signaling pathways, ultimately limiting the durability of clinical responses and contributing to treatment failure, especially in aggressive or advanced-stage cancers [3].

Given these complexities, there is a critical need for innovative therapeutic strategies capable of targeting not only the molecular drivers of tumorigenesis but also the mechanisms of resistance and tumor adaptation [4]. In this context, the search for new bioactive compounds continues to represent a promising area in cancer therapy [5]. These compounds, either newly discovered or repurposed, often exhibit diverse biological activities, favorable safety profiles, and the potential to modulate multiple targets simultaneously. Natural products, in particular, derived from plants, microorganisms, and marine organisms, continue to serve as a rich source of structurally unique and biologically potent agents [6].

The Special Issue “Bioactive Compounds in Cancers” presents recent advances in the identification, characterization, and therapeutic application of such compounds. The contributions span a wide range of approaches, from mechanistic studies and structural optimization to formulation strategies aimed at improving bioavailability and efficacy. This Special Issue brings together a collection of eight contributions that collectively underscore the therapeutic potential of bioactive compounds in various cancer models. The studies can be broadly grouped into three thematic areas: natural compounds and derivatives, synthetic molecules and hybrids, and nanoparticle-based strategies.

Within the realm of natural compounds, research articles explored the anticancer potential of plant-derived or naturally occurring molecules. Gnanamony et al. showed that pomiferin, a natural isoflavone, induced antiproliferative and pro-death effects in high-risk neuroblastoma cells by engaging multiple cell death pathways. It induced ferroptosis through GPX4 inhibition and elevated lipid peroxidation, and also disrupted autophagic processes. In MYCN-amplified LAN5 cells, pomiferin promoted gasdermin E cleavage, indicating pyroptosis, while the lack of changes in phosphorylated MLKL suggested that necroptosis was not involved. Overall, pomiferin induces multiple programmed cell death mechanisms in high-risk NB cells, highlighting its potential to overcome resistance to conventional chemotherapy [Contribution 1]. Similarly, the study on nor-triterpenes from Celastraceae species developed by Reyes et al. identified phenolic derivatives with promising cytotoxic effects against human cancer cell lines, with apoptosis being the main mechanism of cell death observed in HeLa cervical cancer cells. To further investigate their mode of action, the compounds were tested in a drug-sensitive *Saccharomyces cerevisiae* strain, where zeylasteral and 7α-hydroxy-blepharodol demonstrated marked activity in dose–response assays. Halo assays excluded oxidative stress and mitochondrial dysfunction as contributors to their cytotoxicity. Additionally, structure–activity relationship analysis provided insights into the molecular features influencing biological activity. These findings underscore the therapeutic potential of this class of triterpenes and support their further investigation [Contribution 2].

In the category of synthetic and hybrid molecules, contributions focused on the design and optimization of novel compounds with enhanced anticancer properties. Szczepański et al. identified rhodanine–piperazine hybrids as multitarget inhibitors of VEGFR, EGFR, and HER2, demonstrating their potential as anti-breast cancer agents. Additionally, novel hydroquinone-chalcone-pyrazoline derivatives exhibited notable anticancer activity and showed favorable docking interactions with relevant molecular targets. In particular, molecular docking and dynamics simulations confirmed the stable binding of the most promising compounds to HER2, VEGFR, and EGFR, reinforcing the potential of rhodanine–piperazine hybrids as promising candidates for further development, especially for HER2-positive breast cancer [Contribution 3]. A novel series of antitumor hybrid compounds were developed by Maldonado et al. through structural modifications of benzohydroquinone derivatives to enhance antiproliferative activity. These compounds showed notable cytotoxic effects in breast and colorectal cancer cell models. The incorporation of specific functional groups improved their anticancer potential. Molecular docking indicated strong interactions with key kinases involved in carcinogenic pathways, and ADME analysis suggests favorable drug-like properties, supporting their potential for further preclinical evaluation [Contribution 4]. Xanthone–amino acid conjugates were synthesized and evaluated by Barbosa et al., demonstrating promising antitumor activity against various human cancer cell lines, including melanoma, breast, colon, and lung cancers [Contribution 5]. The compound BP-M345, a diarylpentanoid, also emerged as a strong candidate for further development due to its potent antimitotic activity. It induces apoptotic cell death by disrupting mitotic spindle assembly and causing mitotic arrest. Building on this, a series of BP-M345 analogs were synthesized and tested by Moreira et al. in three human cancer cell lines to investigate structure–activity relationships and improve antimitotic efficacy [Contribution 6].

A distinct approach was provided by Kot et al. on lanthanide-doped nanoparticles (GdVO_4_:Eu^3+^ and LaVO_4_:Eu^3+^), which exacerbated oxidative stress in fibroblast cells, exposed to hydrogen peroxide or *tert*-butyl hydroperoxide, at concentrations that are otherwise non-toxic to untreated cells. This effect occurs following nanoparticle internalization and is linked to the activation of caspase-3 and caspase-9, suggesting induction of the intrinsic, mitochondria-dependent apoptotic pathway. The observed apoptosis is driven by excessive ROS production, calcium overload, endoplasmic reticulum stress (via JNK signaling), and DDIT3 expression. These results suggest that GdVO_4_:Eu^3+^ and LaVO_4_:Eu^3+^ nanoparticles can enhance oxidative damage in compromised cells and may hold potential as anticancer agents [Contribution 7].

Complementing the original research articles, this Special Issue also features a comprehensive review by Duda-Madej et al. on the natural alkaloids berberine, sanguinarine, and chelerythrine, highlighting their mechanisms of action and therapeutic potential against colorectal and gastric cancers [Contribution 8].

Taken together, the studies presented in this Special Issue underscore the multifaceted nature of cancer and the critical need for integrative approaches to therapy. The complex interplay of genetic, epigenetic, and environmental factors driving tumor heterogeneity and treatment resistance demands that new therapeutic strategies move beyond single-target agents to embrace multi-targeted, combinatorial, and precision medicine frameworks. Bioactive compounds, whether natural, synthetic, or nanoengineered, offer a rich and versatile arsenal to tackle cancer’s complexity by engaging multiple cellular pathways simultaneously and potentially circumventing resistance mechanisms. However, harnessing their full potential requires not only rigorous mechanistic understanding but also advancements in drug delivery, bioavailability, and safety profiling. This Special Issue highlights the promising progress made in these areas, yet also reminds us that cancer treatment must be viewed holistically—integrating molecular insights, innovative chemistry, and clinical translation—to ultimately improve patient outcomes. Continued interdisciplinary collaboration and translational research will be key to transforming these promising compounds into effective, accessible therapies that address the evolving challenges of cancer worldwide.
